# Sp1 Mediates the Constitutive Expression and Repression of the *PDSS2* Gene in Lung Cancer Cells

**DOI:** 10.3390/genes10120977

**Published:** 2019-11-27

**Authors:** Lanyue Hu, Quanmei Chen, Yitao Wang, Na Zhang, Peixin Meng, Tong Liu, Youquan Bu

**Affiliations:** 1 Department of Biochemistry and Molecular Biology, Chongqing Medical University, Chongqing 400016, China; hulycqmu@163.com (L.H.); qmchen@cqmu.edu.cn (Q.C.); 190372@cqmu.edu.cn (Y.W.); zhangnayyl@163.com (N.Z.); 15648161031@163.com (P.M.); 2017110426@stu.cqmu.edu.cn (T.L.); 2 Molecular Medicine and Cancer Research Center, Chongqing Medical University, Chongqing 400016, China

**Keywords:** *PDSS2*, promoter, transcriptional regulation, Sp1

## Abstract

Prenyl diphosphate synthase subunit 2 (PDSS2) is the first key enzyme in the CoQ_10_ biosynthesis pathway, and contributes to various metabolic and nephritic diseases. It has been reported that *PDSS2* is downregulated in several types of tumors and acts as a potential tumor suppressor gene to inhibit the proliferation and migration of cancer cells. However, the regulatory mechanism of *PDSS2* expression remains elusive. In the present study, we first identified and characterized the *PDSS2* promoter region. We established four different luciferase reporter constructs which mainly cover the 2 kb region upstream of the *PDSS2* gene transcription initiation site. Series luciferase reporter assay demonstrated that all four constructs have prominent promoter activity, and the core promoter of *PDSS2* is mainly located within the 202 bp region near its transcription initiation site. Transcription factor binding site analysis revealed that the *PDSS2* promoter contains binding sites for canonical transcription factors such as Sp1 and GATA-1. Overexpression of *Sp1* significantly inhibited *PDSS2* promoter activity, as well as its endogenous expression, at both mRNA and protein levels in lung cancer cells. Site-directed mutagenesis assay further confirmed that the Sp1 binding sites are essential for proximal prompter activity of *PDSS2*. Consistently, a selective Sp1 inhibitor, mithramycin A, treatment repressed the *PDSS2* promoter activity, as well as its endogenous expression. Chromatin immunoprecipitation (ChIP) assay revealed that Sp1 binds to the *PDSS2* promoter in vivo. Of note, the expression of *Sp1* and *PDSS2* are negatively correlated, and higher *Sp1* expression with low *PDSS2* expression is significantly associated with poor prognosis in lung cancer. Taken together, our results strongly suggest the essential role of Sp1 in maintaining the basic constitutive expression of *PDSS2*, and the pathogenic implication of Sp1-mediated *PDSS2* transcriptional repression in lung cancer cells.

## 1. Introduction

Coenzyme Q 10 (CoQ_10_) is an essential compound of the mitochondrial respiratory chain, and it serves as an electron carrier that delivers electrons from complexes I or II to complex III [1]. CoQ_10_ is presented at all cell membranes and serves as one of the most potent lipophilic antioxidants [2]. It is also crucial in biosynthesizing pyrimidine nucleoside, modulating mitochondrial uncoupling proteins, and regulating cellular apoptosis [3]. Moreover, CoQ_10_ is closely related to the development of malignant tumors. Previous researches showed that the concentration of CoQ_10_ in serum from patients with malignant tumors, such as lung cancer, breast cancer, and cervical cancer, is significantly lower than that of patients without malignant tumors. A low level of CoQ_10_ in patients was positively correlated with poor prognosis [4,5,6]. Another study also suggested that CoQ_10_ can inhibit malignant tumor cell invasion with its antioxidant capacity [7].

The human gene of Prenyl diphosphate synthase subunit 2 (*PDSS2*) is located in chromosome 6q21 and composed of eight exons and seven introns, approximately 30 kb in length. PDSS2 is the first key enzyme in the CoQ_10_ biosynthesis pathway [8]. It has been reported that some *PDSS2* mutations cause CoQ_10_ deficiency and contribute toward metabolic and nephritic diseases [1,9,10]. Recently, the potential of *PDSS2* as a tumor suppressor gene has been investigated, and it has been reported that *PDSS2* is downregulated in gastric cancer, liver cancer, and melanoma, and its downregulation is closely related to tumor stage and grade, suggesting that *PDSS2* may be a new potential tumor suppressor gene [11,12,13]. *PDSS2* also has a decreased expression and tumor-suppressing activity in human lung cancer cells [14].

However, the regulatory mechanism of the human *PDSS2* gene remains unknown, and the promoter region of the human *PDSS2* gene has not yet been identified. In the present study, we have, for the first time, identified the promoter region of the human *PDSS2* gene and found that Sp1 is an important transcription regulator of the *PDSS2* gene. Sp1-mediated *PDSS2* transactivation is implicated in the pathogenesis of lung cancer. This study thus provides the basis for further study of *PDSS2* gene regulation, as well as its functional roles in various biological processes.

## 2. Materials and Methods

### 2.1. Cell Culture, Transient Transfection, and Mithramycin A Treatment

Human Lung cancer cell lines, A549 and H1299, were cultured in DME-F12 for A549 or RPMI 1640 for H1299 medium containing 50 units/mL penicillin, 50 mg/mL streptomycin, and 10% (v/v) FBS (Invitrogen, Carlsbad, CA, USA)). The cells were routinely maintained in humidified atmosphere containing 5% CO_2_ at 37 °C. Mithramycin A (MitA) was purchased from Sangon Biotech (Shanghai, China) and dissolved in DMSO (Sigma-Aldrich, St. Louis., MO, USA). For transient transfection, cells were seeded at a density of 2 × 10^5^ cells/well in 12-well culture plate and then incubated overnight, followed by transient transfection with the indicated plasmids using Neofect DNA^®^ transfection reagent (Neofect biotech, Beijing, China) according to the manufacture protocol. *Sp1* expression plasmid, pcDNA-HA-*Sp1*, was a kind gift from Dr. Jingde Zhu of the Shanghai Cancer Research Institute. The indicated luciferase reporter constructs were transiently co-transfected, the cells were subjected to luciferase assay 48 h after transfection. Twenty-four hours after transfection, MitA (100 nM) was added to the cells. Twenty-four hours after MitA treatment, cells were collected and subjected to further analysis.

### 2.2. Reporter Plasmid Constructions

To assay the promoter activity of the *PDSS2* gene, the luciferase reporters P2031 (−1768/+263), P764 (−501/+263), P464 (−201/+263), and P202 (−201/+1) were established based on the firefly luciferase promoter-less vector pGL3-Basic using the seamless cloning kit (Novorec ^®^PCR NR001, Novoprotein, Shanghai, China). The primer sequences and restriction enzymes are listed in Table S1. Nucleotide sequences of the cloned DNA fragments were confirmed by direct DNA sequencing.

### 2.3. Site-Directed Mutagenesis

The luciferase reporters P202M1, P202M2, P202M3, and P202M4 were generated by a site-directed mutagenesis kit (Toyobo, Japan) on the basis of the indicated parental construct P202 (−201/+1) according to the manufacturer’s instruction. For M1 mutant, the putative Sp1 binding site of CACTCGCCGCCCACAAC at −173 bp was changed into CACTCGCCGTAGACAAC (underlined means changed Nucleotides). For M2 mutant, the putative Sp1 binding site of CAGAGCGGCGGGGTGGG at −126 bp was changed into CAGAGCGAATTTTTGGG. For M3 mutant, the putative Sp1 binding site of AAAGCACCGCCCCTGCGG at −64 bp was changed into AAAGCATAGAATCTGCGG. For M4 mutant, the putative Sp1 binding site of TGCGGGGGCGTTCTCGG at −77 bp was changed into TGCGGGTTAGTTCTCGG. All of the mutations were verified by direct DNA sequencing. The primer sequences are listed in Table S1.

### 2.4. Luciferase Reporter Assay

Luciferase reporter assay was used to assess the promoter activity of the *PDSS2* gene. The assay was conducted as described previously [15,16]. In brief, cells were seeded in 12-well plates in triplicate, and then transfected with the internal control vector of renilla luciferase pRL-TK reporter (10 ng) (Promega, Madison, WI, USA), and the firefly luciferase reporter plasmids containing the corresponding *PDSS2* promoter fragments (100 ng) using Neofect DNA^®^ transfection reagent. Forty-eight hours later, cells were lysed, and luciferase activity was determined using the Dual-Luciferase assay system (Promega, Madison, WI, USA).

### 2.5. DNA Sequence Alignment and Database Analysis

The mRNA and genomic sequences of *PDSS2* were downloaded from GeneBank and the UCSC database. Transcription factor binding sites were predicted using online software JASPAR, PROMO, and MatInspector. Alignment for the *PDSS2* promoter sequences from multiple species was conducted using DNAMAN and Clustal Omega.

### 2.6. Chromatin Immunoprecipitation

ChIP was performed with the EZ ChIP™ Chromatin Immunoprecipitation kit (Upstate, Lake Placid, NY, USA) as described previously [15,16]. The sequences of the primers are given in Table S2, and the Sp1 antibody was purchased from Upstate.

### 2.7. RT-PCR

Real time quantitative PCR (RT-PCR) was conducted as described previously [17]. Briefly, total RNA was isolated from the indicated cells using the Total RNA Kit I (Omega Bio-Tek, Norcross, GA, USA), cDNA was synthesized from 1 μg of total RNA using the PrimeScript 1st Strand cDNA Synthesis Kit (Takara, Tokyo, Japan), and RT-PCR was performed using the SYBR^®^ Premix Ex TaqTM (Perfect Real Time, Takara, Tokyo, Japan). The sequences of the primers are offered in Table S2.

### 2.8. Western Blotting

Western blotting was conducted as described previously [15,16]. Briefly, cells were collected and lysed with RIPA buffer supplemented with protease inhibitor Cocktail (Biotool, Houston, TX, USA). The total proteins were determined using the bicinchoninic acid (BCA) protein assay kit (Thermo Scientific, Beijing, China) and then subjected to SDS-PAGE and immunoblotting. The blots were visualized by enhanced chemiluminescence (ECL, Bio-Rad Laboratories, Hercules, CA, USA). The primary antibodies were anti-Sp1 (Bimake, Shanghai, China, dilution 1:1500, with secondary anti-rabbit antibodies) and anti-PDSS2 (Santa Cruz Biotechnology, Santa Cruz, CA, USA, dilution 1:200, with secondary anti-mouse antibodies). For the *Sp1* overexpression experiment, *Sp1* expression plasmids and corresponding empty vectors were transiently transfected in cells. Forty-eight hours after transfection, whole cell lysates were prepared and subjected to Western blotting analyses. GAPDH was used as an internal control.

### 2.9. Expression Analysis of Sp1 and PDSS2 in Lung Cancer

The prognostic value of *PDSS2* expression was analyzed using published lung cancer microarray data as described previously [17]. Microarray and patient survival data were downloaded from the public GEO database (GSE13213). The microarray raw data were routinely processed and further used for expression correlation and survival analysis. Receiver operating characteristic curve analysis was conducted to obtain a rational cut-off point [17].

### 2.10. Statistical Analysis

All data were statistically analyzed using SPSS 16.0 software (SPSS INC., Chicago, IL, USA). The luciferase reporter gene activity was compared between two groups using the unpaired *t*-test. *p* < 0.05 was considered statistically significant.

## 3. Results

### 3.1. Genomic Structure of the PDSS2 Gene Locus

**Figure 1:**
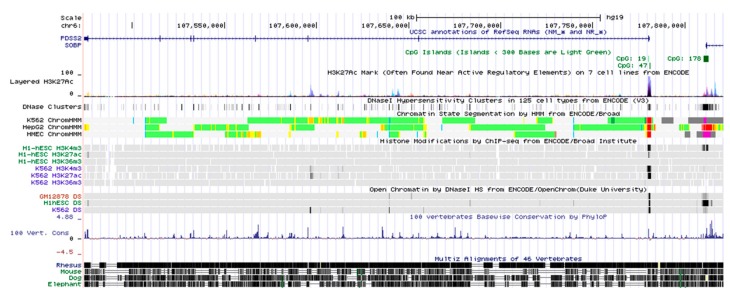
Schematic diagram of genomic organization and chromatin state of the human *PDSS2* gene locus. The genomic region of the *PDSS2* gene (chr6:107, 441, 132-107, 901, 660, human species genomic assembly version, GRCh37/hg19) is schematically represented with the indicated tracks, including exon-intron structure, H6K27Ac markers, DNase I hypersensitive clusters, histone modifications, and vertebrates conservation. The chromatin state was indicated as active promoter (bright red), strong enhancer (orange), insulator (blue), transcriptional elongation (deep green), and weakly transcribed region (light green).

To analyze the genomic organization of the human *PDSS2* gene, as well as its chromatin state, we deeply retrieved and mined the UCSC genome database (http://genome.ucsc.edu/). As shown in Figure 1, the *PDSS2* gene is located at human chromosome 6p21 and contains eight exons interspaced by seven introns. According to the ENCODE histone modification data, the transcription elongation hallmark of H3K36me3 was enriched within the whole *PDSS2* gene body. In line with this evidence, ChromHMM chromatin state data mining also suggested that the *PDSS2* gene was actively transcribed in cells. Of note, the first exon and the 5′-region of first intron of the *PDSS2* gene contained a classic CpG island, and was marked with DNase I hypersensitivity and H3K4me3 (a hallmark of transcriptional initiation), highly suggesting that the *PDSS2* gene promoter is located near its first exon region.

### 3.2. Identification of the PDSS2 Promoter Region

**Figure 2:**
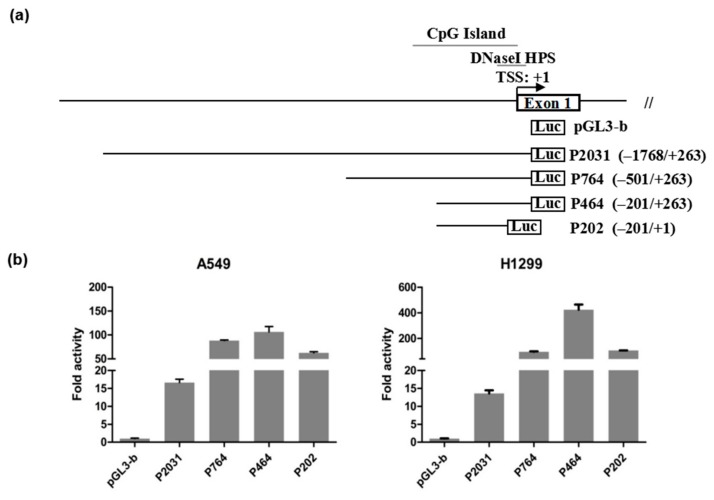
Identification of the *PDSS2* promoter region. (**a**) Schematic diagram of the *PDSS2* gene promoter reporter constructs. The constructs are named as P-length (start position/end position). The open box is shown as the first exon of *PDSS2*, the positions relative to the major *PDSS2* transcription start site (+1) are indicated. The positions of the putative DNaseI HPS and CpG islands are also retrieved. (**b**) Luciferase assay of the *PDSS2* promoter reporter constructs. Data obtained from a representative of at least three independent experiments are shown as fold induction compared to the activity of cells transfected with the empty reporter vector, pGL3-b. The results are presented as the mean and standard deviation (SD) of triplicate results from a representative experiment (*p* < 0.01).

To determine the potential proximal promoter region of the *PDSS2* gene, a variety of the *PDSS2* gene promoter fragments that differed in length were inserted into the firefly luciferase reporter vector, pGL3-Basic. In total, we generated four luciferase reporter constructs, including P2031 (−1768/+263), P764 (−501/+263), P464 (−201/+263), and P202 (−201/+1) (Figure 2a, Table S1). As shown in Figure 2b, the luciferase activities of all of the four constructs were significantly enhanced compared to pGL3-basic group in two lung cancer cell lines, H1299 and A549. Among the four constructs, the activity of P464 (−201/+263) was the highest, while the activity of the longest construct, P2031 (−1768/+263), was the lowest. The activity of P764 (−501/+263) was close to that of P202 (−201/+1). It is noteworthy that the activity of P764 (−501/+263) was higher than that of P2031 (−1768/+263), suggesting that the region from −1768 to −501 bp might contain silencer elements. The activity of the shorter P464 (−201/+263) was higher than that of P764 (−501/+263), suggesting that there might be silencer elements in the −501 to −201 bp region. The activity of the shortest P202 (−201/+1) was lower than that of P464 (−201/+263), suggesting that there may be enhancer elements in the +1 to +263 bp region. Nevertheless, the shortest P202 (−201/+1) shows robust activity, suggesting that the *PDSS2* core promoter region is harbored in this 201bp fragment.

### 3.3. Sequence and Homology Analysis of the PDSS2 Promoter Region

**Figure 3:**
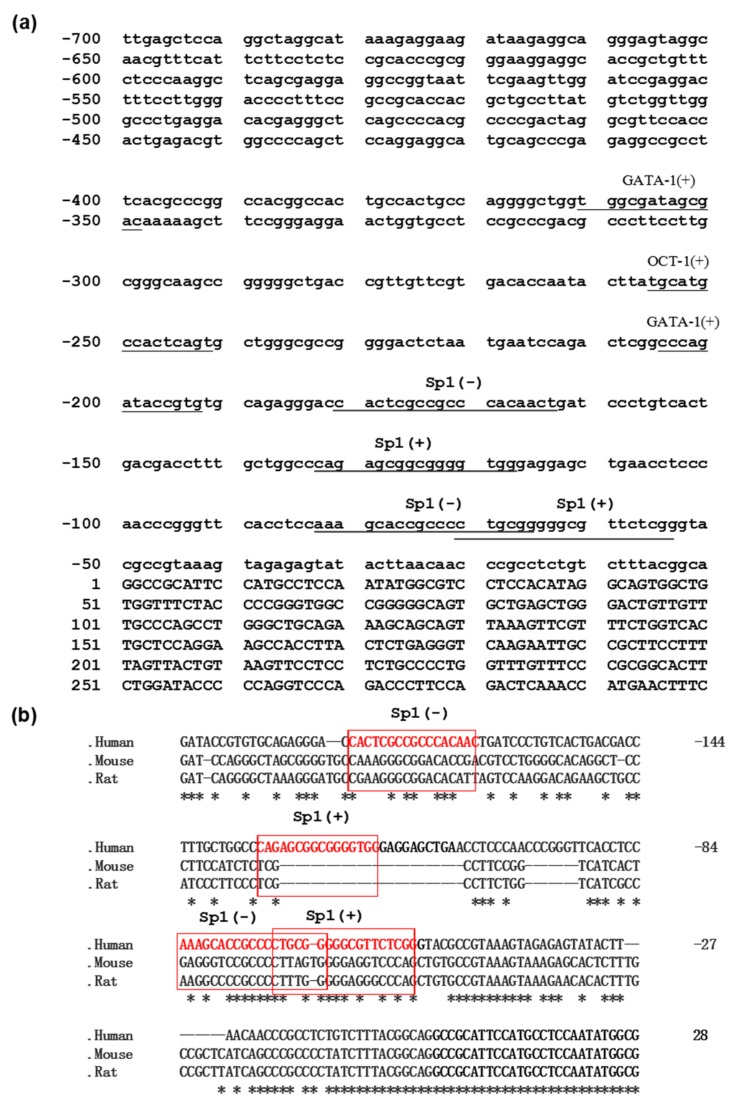
Nucleotide sequence homology analysis of the promoter region of the *PDSS2* gene. (**a**) The nucleotide sequences of putative transcription factor binding sites are underlined. The positions relative to the major *PDSS2* transcription start site (+1) are indicated. (**b**) Sequence alignment of the nucleotide sequences of the *PDSS2* gene promoters from human, mouse, and rat species was performed by DNAMAN software. The potential Sp1 binding sites are boxed. The identical bases among human, mouse, and rat species are marked with stars (*).

To further explore the potential cis-acting regulatory elements for the *PDSS2* gene, we employed multiple software to analyze transcription factor binding sites for the *PDSS2* promoter. As indicated in Figure 3a, the results show that the *PDSS2* promoter contains several consensus binding sites for canonical transcription factors such as Sp1. In addition, homology analysis revealed that the *PDSS2* promoter sequence is evolutionarily conserved across species of human, mouse, and rat (Figure 3b). Since four potential Sp1 binding sites were evolutionarily well conserved among the three species, it is highly suggestive that Sp1 might be a critical direct regulator for *PDSS2* gene transcription.

### 3.4. Sp1 Inhibits PDSS2 Expression

**Figure 4:**
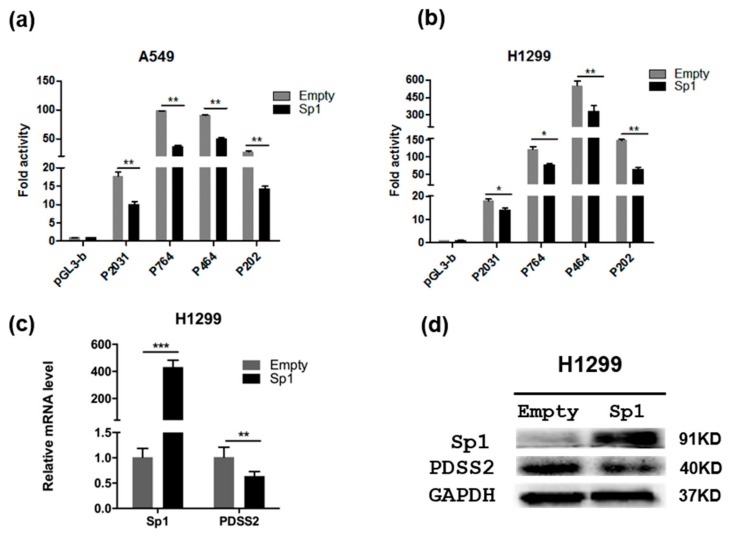
Sp1 inhibits *PDSS2* gene expression. Overexpression of *Sp1* inhibits *PDSS2* promoter activity, both in A549 (**a**) and H1299 (**b**) cells. Exogenous expression of *Sp1* represses endogenous *PDSS2* expression, both at mRNA levels (**c**) and protein levels (**d**). GAPDH was used as an internal control (* 0.01 < *p* < 0.05, ** *p* < 0.01, *** *p* < 0.001).

To determine whether Sp1 regulates *PDSS2* gene transcription, *Sp1* expression plasmid were co-transfected with the indicated *PDSS2* luciferase reporter constructs, both in A549 and H1299 cells. The luciferase reporter assay showed that overexpression of *Sp1* significantly inhibited the luciferase activity of all of the four reporter constructs with various degrees when compared with the empty vector transfected group (Figure 4a,b). In fact, multiple typical Sp1 binding sites existed in all of the four selective *PDSS2* promoter fragments (Figure 3). Next, we further determined whether Sp1 represses the endogenous transcription of *PDSS2* in cells. As shown in Figure 4c,d, exogenous overexpression of *Sp1* resulted in significant repression of the endogenous *PDSS2* expression, both at mRNA and protein levels. Therefore, these results clearly indicate that Sp1 represses the expression of the *PDSS2* gene.

### 3.5. Functional Analysis of Sp1 Binding Sites in the PDSS2 Core Promoter

**Figure 5:**
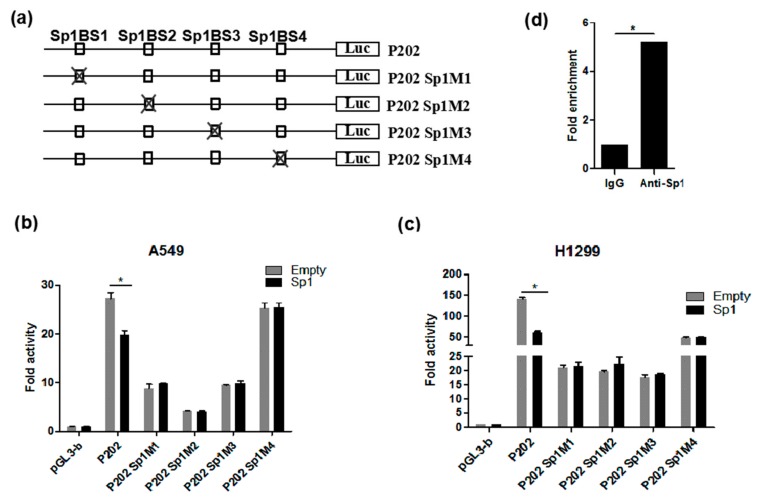
Functional analysis of Sp1 binding sites in the *PDSS2* core promoter region. (**a**) Schematic diagram of site-directed mutagenesis of Sp1 binding sites in the *PDSS2* core promoter. The four potential Sp1 binding sites are indicated as open boxes (Sp1CBS1, SpCBS2, Sp1CBS3, and Sp1CBS4). The indicated point mutation is denoted by a cross. (**b**,**c**) Luciferase reporter assays. The indicated luciferase reporter constructs were introduced into A549 (**b**) and H1299 (**c**) cells, and luciferase activities were determined as described in Figure 2 (* 0.01 < *p* < 0.05). (**d**) ChIP assay. Chromatin fragments were prepared from H1299 cells and immunoprecipitated with anti-Sp1 antibody or control IgG. The precipitated DNA was then amplified by real-time PCR with primers directed to the Sp1 binding sites in the *PDSS2* core promoter region.

To further verify the functional involvement of Sp1 binding sites in the *PDSS2* core promoter region, point mutations were introduced to disrupt each of the four putative Sp1 binding sites of the *PDSS2* core promoter, respectively. The corresponding luciferase reporter constructs were designated as P202M1, P202M2, P202M3, and P202M4, respectively (Figure 5a). As shown in Figure 5b,c, disruption of each of the four Sp1 binding sites—especially the first three sites—resulted in significant decreased *PDSS2* core promoter activity in both H1299 and A549 cells. Intriguingly, disruption of the fourth Sp1 binding site (Sp1BS4) led to significant repressed promoter activity in H1299 but not A549 cells, suggesting a cell type-dependent mechanism. Of note, disruption of each of the four Sp1 binding sites completely abolished the inhibitory effect of *Sp1* overexpression on *PDSS2* promoter activity. Therefore, these results strongly suggest that all the four Sp1 binding sites in the *PDSS2* core promoter region are not only critical for its constitutive core promoter activity, but also essential for Sp1-mediated repression of *PDSS2* transcription. In addition, Chromatin immunoprecipitation (ChIP) assay demonstrated that Sp1 binds to the *PDSS2* core promoter region in vivo in cells (Figure 5d), suggesting that Sp1 is a bona fide direct regulator for the human *PDSS2* gene.

### 3.6. Sp1 Is Essential for the Constitutive Expression of PDSS2 Gene

**Figure 6:**
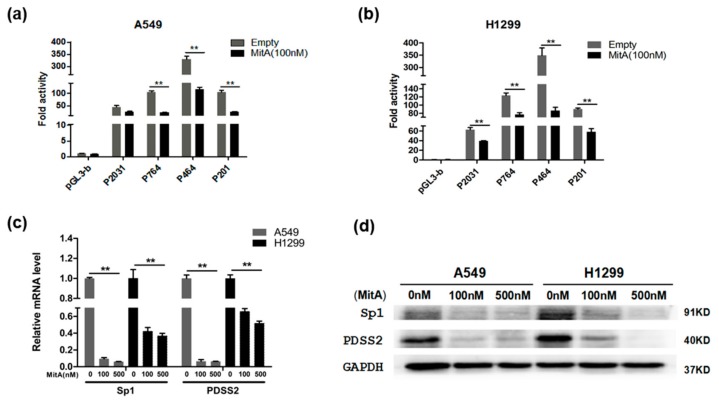
Sp1 is essential for the basic expression of the *PDSS2* gene. The indicated luciferase reporter constructs were transiently co-transfected together with pRL-TK in A549 (**a**) and H1299 cells (**b**). Twenty-four hours after transfection, mithramycin A (MitA, 100 nM) was added. The cells were harvested 24 h after the addition of mithramycin A and subjected to luciferase assay. (**c**,**d**) A549 and H1299 cells were treated with Mithramycin A (100 nM, 500 nM). Twenty-four hours later, cells were harvested, and total RNA and cell lysates were prepared and subjected to qRT-PCR and Western blotting analyses, respectively (** *p* < 0.01).

To further investigate the role of Sp1 in regulating the constitutive expression of the *PDSS2* gene, we chose to utilize a selective Sp1 inhibitor to execute a loss of function experiment of Sp1. As a selective inhibitor, mithramycin A (MitA) is known to bind to GC-rich DNA sequences, displacing Sp1 transcription factor binding to its target promoters, which inhibits their expression [15]. As shown in Figure 6a,b, luciferase reporter assays revealed that MitA treatment resulted in significant decreased promoter activity of the *PDSS2* promoter reporters in A549 and H1299 cells. Furthermore, MitA treatment also led to remarkable repression of endogenous *PDSS2* expression at both mRNA and protein levels in both A549 and H1299 cells (Figure 6c,d). These results are consistent with the observation of decreased promoter activity caused by disruption of Sp1 binding sites in the *PDSS2* core promoter region (Figure 5a–c), highly suggesting that Sp1 is an essential transcriptional regulator for the basic constitutive expression of the *PDSS2* gene in cells.

### 3.7. Sp1-Mediated PDSS2 Expression Is Associated with Poor Prognosis

**Figure 7:**
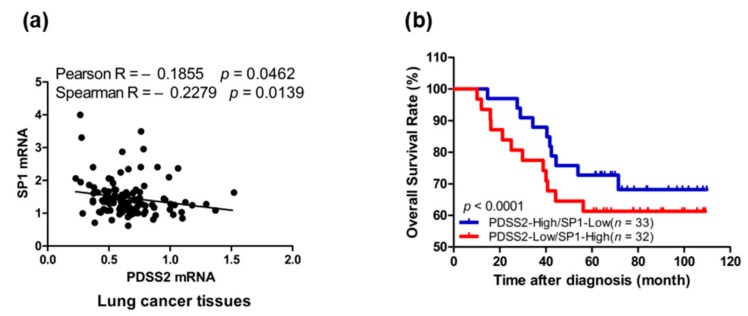
Clinical significance of Sp1-mediated *PDSS2* repression in lung cancer. (**a**) Correlation analysis between *Sp1* and *PDSS2* expression in a lung cancer cohort (GEO database, GSE13213). (**b**) Survival analysis of *Sp1* and *PDSS2* expression in the same lung cancer cohort.

Finally, we asked whether Sp1-mediated *PDSS2* repression exists and represents clinical significance in lung cancer. To this end, the correlation of *Sp1* and *PDSS2* expression in lung cancer tissues from a lung cancer cohort was analyzed. The results show that the expression of *Sp1* is negatively correlated to that of *PDSS2* in lung cancer tissues (Figure 7a). Furthermore, Kaplan–Meier survival analysis revealed that patients of lung cancer with lower expression of *Sp1* and higher expression of *PDSS2* exhibited the best disease-free survival, rather than higher expression of *Sp1* and lower expression of *PDSS2* (Figure 7b).

## 4. Discussion

Previous investigations clearly indicated that the expression levels of *PDSS2* are remarkably downregulated in several type of malignant tumors compared with their normal counterparts, including non-small cell lung cancer, primary melanoblastoma, gastric cancer, and liver cancer [11,12,18,19]. Gain-of-function studies revealed that exogenous overexpression of *PDSS2* prominently inhibits tumor cell growth and invasion, highly suggesting that *PDSS2* might act as a novel potential tumor suppresser gene and serve as a potential therapeutic target for cancers. Therefore, elucidation of its expression regulation mechanism is of great significance for investigating its molecular behavior, as well as designing novel therapeutic strategy for cancers.

Up to now, all the reports regarding the role of *PDSS2* in cancers focus on its expression profiling and functional analysis. Our present study is the first to investigate the regulatory mechanism of this cancer-related gene. We have successfully identified the *PDSS2* promoter and located its core promoter in a short 202 bp region near its transcription start site. Functional analysis revealed that the Sp1 transcription factor is a critical regulator for the constitutive expression, as well as its repressed expression, of *PDSS2* in lung cancer cells. The increased *Sp1* expression was observed in several types of cancers, including lung cancer, pancreatic cancer, colon cancer, gastric cancer, and glioma cancer, and is associated with a poor prognosis in some cancers [20,21,22,23]. Notably, studies also showed that Sp1 plays a role in promoting cancer cell proliferation, angiogenesis, migration, and invasion through regulating a variety of genes involved in tumorigenesis, and thus exhibits pro-oncogenic function, suggesting Sp1 is a potential target for the treatment of cancers [24]. Our present study revealed *PDSS2* as a novel target gene of Sp1. PDSS2 is known to be the first key enzyme in the pathway of CoQ_10_ biosynthesis. We thus speculate that Sp1 could contribute to the development of malignant tumors through decreasing CoQ_10_ biosynthesis by regulating *PDSS2*. This regulatory axis is of great significance in the biomedical field and is currently under deep investigation in our lab.

It is worth noting that multiple GC boxes, along with high-scoring CpG islands, were observed in the *PDSS2* promoter region, suggesting that epigenetic modifications are involved in regulating the expression of the *PDSS2* gene, such as methylation modification. Hypermethylation of the *PDSS2* promoter was detected in hepatocellular carcinoma cells and gastric cancer cells with low *PDSS2* expression, and the expression of *PDSS2* was re-activated after demethylation [12,18]. Therefore, we deduced that Sp1 might recruit DNMT1 to promote DNA methylation in the *PDSS2* promoter region, resulting in repressed expression of *PDSS2* transcription in cancer cells.

In addition, we also found that the *PDSS2* promoter region contains canonical OCT-1 (Octamer binding transcription factor 1) and GATA-1 binding sites. It has been reported that OCT-1 promotes cancer cell proliferation, migration, and invasion, such as in lung cancer, prostate cancer, and cervical cancer, and its high expression is closely related to the poor prognosis of patients [25,26,27,28]. GATA-1, the founding member of the GATA family of transcription factors, typically binds to DNA-specific nucleic acid sequence sites A/T GATA A/G, and stimulates or suppresses the expression of its downstream target genes [29,30]. The expression of GATA-1 has been shown to be upregulated in breast cancer and repress the E-cadherin transcription by binding to the E-cadherin promoter and recruiting HDAC3/4. PAK5-mediated GATA-1 phosphorylation regulates EMT in breast cancer cells. GATA-1 associates with the histone methyltransferase SET7 to promote VEGF transcription and breast tumor angiogenesis [31,32,33]. GATA-1 promotes cell proliferation, migration, and invasion via activating the PI3K/AKT signaling pathway in colorectal cancer [34]. As important transcriptional regulators, OCT-1 and GATA-1 are involved in the regulation of a variety of physiological and pathological processes, including the development of cancers. Therefore, future works are needed to investigate whether the transcription of *PDSS2* is regulated by OCT-1 or GATA-1.

In conclusion, our preset study identified the promoter region of the *PDSS2* gene for the first time and demonstrated that Sp1 transcription factor could directly regulate the transcription of *PDSS2*, which not only lays a solid foundation for further elucidation of the *PDSS2* regulation mechanism and molecular behavior, but also contributes to further analysis of the biological function of *PDSS2* in the development of cancer.

## Supplementary Materials

All the supplementary data for this article are available online at https://www.mdpi.com/2073-4425/10/12/977/s1. Table S1, The primer sequences for PDSS2 promoter reporter construction; Table S2, The primer sequences for RT-PCR and ChIP.
