# Supplementary material for: Sp1 Mediates the Constitutive Expression and Repression of the PDSS2 Gene in Lung Cancer Cells
Source: Genes (Basel). 2019 Nov 27;10(12):977. doi: 10.3390/genes10120977 (PMC6947312; doi:10.3390/genes10120977)
Supplement: Supplementary file 1 [file genes-10-00977-s001.pdf]

Supplementary Materials:

Table S1 The primer sequences for *PDSS2* promoter reporter construction

Constructs	Primer sequences	Restriction site
P2031(-1768/+1263)	F:5'- GGGGTACCAGGGCATAGACTGTGTTTGATAC -3'	*Kpn* I site
	R:5'- CTAGGCTAGCTGGGGGTATCCAGAAGTGCCG -3'	*Nhe* I site
P764(-501/+263)	F:5'- GGCCCTGAGGACACGAGGGC -3'	*Kpn* I site
	R:5'- GGTACCTATCGATAGAGAAATGTTCTGGC -3'	*Nhe* I site
P464(-201/+263)	F:5'- GATACCGTGTGCAGAGGGACCA -3'	*Kpn* I site
	R:5'- GGTACCTATCGATAGAGAAATGTTCTGGC -3'	*Nhe* I site
P202(-201/+1)	F:5'- GCTAGCCCGGGCTCGAGATCTGCGATCT-3'	*Kpn* I site
	R:5'- TGCCGTAAAGACAGAGGCGGGTTGTTAAG -3'	*Nhe* I site
PDSS2-P202M1	F:5'- ACAACTGATCCCTGTCACTGACGACC -3'	*Kpn* I site
	R:5'- CTACGGCGAGTGGTCCCTCTGCAC -3'	*Nhe* I site
PDSS2-P202M2	F:5'- AATTTTTGGGAGGAGCTGAACCTCCC -3'	*Kpn* I site
	R:5'- CGCTCTGGGCCAGCAAAGGTCGTCAG -3'	*Nhe* I site
PDSS2-P202M3	F:5'- TAGAATCTGCGGGGGCGTTCTCGGGT -3'	*Kpn* I site
	R:5'- TGCTTTGGAGGTGAACCCGGGTTGGG -3'	*Nhe* I site
PDSS2-P202M4	F:5'- TTCTCGGGTACGCCGTAAAGTAGAG -3'	*Kpn* I site
	R:5'- CTAACCCGCAGGGGCGGTGCTTTG -3'	*Nhe* I site

Table S2 The primer sequences for *RT-PCR and CHIP*

Gene	Primer sequences
GAPDH(*RT-PCR*)	F:5'- ACCTGACCTGCCGTCTAGAA -3'
	R:5'- TCCACCACCCTGTTGCTGTA -3'
Sp1(*RT-PCR*)	F:5'- GTGGAGGCAACATCATTGCTG -3'
	R:5'- GCCACTGGTACATTGGTCACAT -3'
PDSS2(*RT-PCR*)	F:5'- TCTAGCAAATGCCTGCAATG -3'
	R:5'- TCTGCTCCTTCCAAGTCGAT -3'
PDSS2(*CHIP*)	F:5'- CGCCCACAACTGACCCTGTCA -3'
	R:5'- GAATGCGGCCTGCCGTAAAGAC -3'
